# Gleanings

**Published:** 1893-07

**Authors:** F. H. Lutze

**Affiliations:** Brooklyn, N. Y.


### GLEANINGS.

F. H. LUTZE, M. D., BROOKLYN, N. Y.

During the past few years I have made it a practice to copy whatever peculiar symptoms I saw in the journals and place them in my *Repertory* or *Materia Medica,* where I could readily refer to them when I wished to do so. On looking them over lately I thought the collection valuable enough to offer them to the profession in general, and the result is before you.

It would have been a pleasure to give with each symptom the name of the author from whose article the same had been taken, but as they were copied and written where they would do the most good for myself without any thought of ever publishing them, this is not now possible. In general I may say, however, that nearly all were copied either from the *Medical Advance* or THE HOMŒOPATHIC PHYSICIAN.

### MIND.

Affection of friends, thinks she has lost it. Aurum, Hura.

Despised, thinks he is. Argent-nitr., Lac-can.

Mania acute, produced sooner or later by injuries to brain; satiety of life, must use great self-control to prevent shooting himself. Natr-sulph.

Snakes on back, thinks there are; is frightened and sick. Lac-can.

Strikes forehead with hands. Ars.

— head against wall or floor. Rhus.

Memory defective, has to read it several times to comprehend it. Ambra. (Alumina, Aurum, Carb-v., Lycopod., Rhus-t., Veratr, Zinc).

Memory, remembers easy what he reads. Anacard.

Fear, trembling, restlessness, prostration, cold sweat when about to make a journey or undertaking anything. Ars.

Relaxation, mental and physical; don't want to be spoken to; desires to be alone; lack of mental and physical courage; afraid to appear in public, as to speak or sing. Gels.

Stupor, goes into a, while answering. Baptisia.

— patient answers questions correctly then lapses into a stupor. Arn.

— — passes into a stupor from which it is impossible to arouse him, so profound; pinched countenance. Opium.

Sees words when reading, but cannot attach any meaning to them. Ignat.

Fear of people, likes to be alone. Iod.

Mental depression, as if a cloud had settled over patient; distressed and suspicious without cause. Cimicifuga.

Fright recent, causing hemorrhage. Opium.

Cross and irritable like. Cham., Rumex.

Mad, raving, tearing, and swearing. Valeriana (differs in this from Puls. which is mild and tearful).

Visions on closing eyes. Argent-nit., Bell., Bry., Calc-c., Chin., Ignat., Thuja.

Patient answers very slowly, comprehension sluggish. Sulph.

Mania from injuries to brain; drawing back of neck; spasms of back; melancholia, wants to shoot himself; mental irritability and gloom; violent crushing and gnawing pains at base of brain, as if a dog was gnawing there; sadness and delirium; congestion to head. Natr-sulph.

Fright from report of a gun or thunder. Borax.

### Sensorium.

Sensation of liquids being forced through longitudinal sinus, pressure grows so severe as to produce anxiety, perspiration, and red face; pressure from forehead to vertex. Glonoin.

Sensation as if liquids were moving up and down the thigh, or any other part of body; no pain. Opium.

— — — a fluid were injected paroxysmally in a small blood-vessel, with violent, raging pain from right eye to temporal bone or any other part of body. Cocc-cact.

Vertigo on closing eyes, ceases on opening them. Thuja.

— when urinating only. Hyperic.

— with sensation as waking up.

— everything turns in a circle. Sabadil.

— in air. Merc-iod-flav.

— as if turning in a circle. Argent-nit.

Ants running through body, sensation of; evenings, when lying down, then difficult breathing, is obliged to get up and open window, better fresh air but immediately on lying down the sensations return again. Cann-ind.

Dizzy, the child is, when carried by nurse, seizes hold of nurse, fearing to fall. Gels.

— child seizes hold of nurse, when being lowered in bed. Borax.

Child seizes hold of nurse for fear of being separated. Cuprum.

Answers questions correctly and lapses into a stupor. Arn., Hyos.

Answering, while, goes into a stupor. Baptis.

Stupor profound, impossible to arouse; pinched countenance; involuntary urine and stool; carphalogia, sliding down in bed. Hyos.

Sensation of a ball in inner parts. Ignatia.

Vertigo on turning to the left, and nausea, a fainty sickness. Iod.

Ball, sensation of, in inner parts. Ignat.

— — — — bladder. Lachesis.

Sensation of numbness in outer, and constriction in inner parts. Opium.

— as if parts would burst. Ran-bulb.

— of a tight band around the head and forehead, as if hat were too tight; has to remove it often, but it does not improve it. Sarsap.

Rubs the forehead. Veratr-alb.

Syphilitic destruction of bones of forehead. Aurum.

Headache. Begins morning soon after rising, in forehead; better from pressure of hand. Cinnabar.

— with vomiting, nausea, and high fever. Veratr-vir.

— in small spot over right eye; becomes red and sore; better from hard pressure. Sanguinaria.

— worse jar, noise, moving; pain extends to eyeball; pillow feels hard as a stone. Arnica.

— frontal, with cold sensation, as if cold wind was blowing upon it. Laurocer.

— going to side lain on. Puls., Phos-ac., Calc-ars., Bry.

— aching, severe in left mastoid process, extending upward and downward on moving head. Calc-c.

— better from a thin stool. Æthus., Agar., Corn-c., Lachn., Oxal-ac.

— better from copious flow of urine. Gels.

— on awaking; better after stool. Ptelea.

— undulating mornings. Hip.

— — evenings. Sulph.

— with intense bruised feeling of eyeballs, and abdominal pains. Chionanthus.

— in winter, diarrhœa in summer. Aloe.

— sick, on left side, face pale on that side; constriction of chest and throat. Laches., Amyl-nitros.

— from riding on the cars. Arnica.

— in temples and eyes; better cold; face blue, bloated, eyes turgesced. Bry.

— worse cold. Rhus.

— better lying with head low. Calc-c.

— — — raised. China.

— from overlifting or loss of breakfast. Calc-c.

— pain seats itself in forepart of head on drinking water, and extends into nose. Digit.

— better from slight motion. Ferr-acet.

— violent day and night; worse during scanty sweat; bitter taste; aching in bones as if they would break; yellow, dry skin; vomiting of bile (malarial headache). Eupator-perf.

Headache preceded by blindness; on right side of forehead, throbs with nausea; worse evening, cold air, coughing, rest; better by moderate motion. Gels. And profuse discharge of limpid urine. Gels.

— pain goes around to back of head, preceded by appearance of a zigzag wheel before the eyes, with play of colors, numbing the intellect. Ignat.

— begins with a blur before the eyes; sick headache every eighth day; sharp, cutting pains, change location often. Iris-vers.

— sick; giddiness, vertigo, and vomiting, semicircular and backward movements, rolling over on axis of body. Coccul.

— white, glittering zigzags, serpentine; white glittering at side of visual field, after dinner hyperæsthesia of emotions and special senses, difficulty of thinking and speaking, emotional disturbance, worse from emotion (Clavus), better voiding a quantity of limpid urine. Ignat.

— more vertigo, then Ignatia, as much hyperæsthesia, less emotional excitement, disturbed temper; cause, error in diet. Nux-v.

— flickering roses or round spots before the eyes, vertigo, severe and lasting vomiting, slow pulse, pale face, contracted arteries. Digital.

— obscuration of sight, flickering before the eyes, headache for two days, less on third day, disappears on fourth day; a luminous ball or dark disk pierced by brilliant lightning; vertigo, mental confusion with menstrual derangements, blind headache. Cyclamen.

— sick with blur or misty vapors before the eyes. Iris-vers.

— sick. Bell., Calc-c., Coccul., Ignat., Nux-v., Digit., Cyclamen, Iris-vers., Sepia, Sanguinar., Silicea, Stannum, Paris-quad., Strontia, Zinc, Sulph.

— sees a white star as large as a plate, on looking up (to ceiling) and white, silvery clouds pass over it. Bell.

Headache, nervous of women. Silicea, Menyanthes, Paris-quad., Strontiana.

-- pain ascends from nape of neck to vertex, and thence to supraorbital region; worse, noise, motion, or concussion; better, wrapping head up warmly. Silicea.

— pain ascends from nape of neck over head, it is a bursting pain as if the membranes of the brain were tense, and bursting the skull open; better from pressure. Menyanthes.

— pain ascends from nape of neck overhead, which feels as if immensely large. Paris-quad.

— — — — — — — spreads over head, begins lightly, increases gradually to its greatest intensity, and gradually decreases (Stannum); better wrapping head up warmly. Stontia.

— from loss of breakfast. Calc-c.

— of school girls. Calc-phos., Natr-m., Phos-ac.

Hæmicrania, left side; worse after sleep in the daytime, worse evenings; in paroxysms, severe nausea; worse from constipation, out-door exercise, riding; must keep perfectly quiet. Nux-v.

— as if cord were attached to left eye, drawing it back into head. Paris-quad.

Headache on arising or waking; head is dizzy; heavy and aches, as if he had lain with head too low every morning; can't think; headache, partly dull (empty), partly pressing; a lurching in the head or tearing; worse motion; compressive headache day after day. Phos.

— with hunger. Psorinum, Phos.

— like hammers striking the head from within outward. Psor. (Calc-c., Natr-mur.)

— and vertigo; worse looking fixedly at, or thinking of, a subject. Sabad.

— every seventh day. Sabad., Silicea, Sulph.

Head, want of power to hold up the head. Æthus.

Hydrocephalus, arms below elbows become deathly cold. Arnica.

Throbbing through temples after loss of blood. China.

Pressure in head and oppressive as if apoplexy threatened; better walking. Colch.

Head feels pithy, like cork, and seems to patient to be elongated upward. Graph.

Rolling the head. Helleb., Hyos., Podoph.

Headache from both temples to base of brain; worse from heat; vertex itches during attacks; pain compels her to move about, but motion does not relieve; cold perspiration. Veratr-alb.

Head, congestion to, chest and arms, with cold feet, is obliged to move them constantly. Lil-tig.

### Scalp.

Hair falling out. Aloe, Amm-m., Vinca.

— — — head sore on right side. Amber-gris.

— — — in lumps, leaves bare patches, frontal headache. Aloe.

— — — which looks dead; large accumulation of bran-like scales; great itching of scalp. Amm-m.

— — — bald patches at or near forehead; scalp covered with dry scabs or scales. Ars.

— — — after severe illness or confinement. Carb-v.

— — — with itching of the head; the new hair is dry and breaks. Fluor-ac.

— — — dry hair; rapidly, with much dandruff. Kali-carb.

— — — in circular spots; bald spots behind ears. Phos.

— — — in single spots, and white hair grows there; spots on head oozing moisture; hair matting together. Vinca-minor.

Scalp: hair tangles easy, like a rat's nest, from scalp to tip. Psorin.

— — — — tips only. Borax.

